# Combination of Electroacupuncture and Grafted Mesenchymal Stem Cells Overexpressing TrkC Improves Remyelination and Function in Demyelinated Spinal Cord of Rats

**DOI:** 10.1038/srep09133

**Published:** 2015-03-16

**Authors:** Ying Ding, Rong-Yi Zhang, Bing He, Zhou Liu, Ke Zhang, Jing-Wen Ruan, Eng-Ang Ling, Jin-Lang Wu, Yuan-Shan Zeng

**Affiliations:** 1 Department of Histology and Embryology, Zhongshan School of Medicine, Sun Yat-sen University, Guangzhou, Guangdong 510080, China; 2 Key Laboratory for Stem Cells and Tissue Engineering Ministry of Education, Sun Yat-sen University, Guangzhou, Guangdong 510080, China; 3 Institute of Spinal Cord Injury, Sun Yat-sen University, Guangzhou, Guangdong 510127, China; 4 Co-innovation Center of Neuroregeneration, University, Nantong, Nantong, Jiangsu 226011, China; 5 Department of Acupuncture, the 1st Affiliated Hospital, Sun Yat-sen University, Guangzhou, Guangdong 510080, China; 6 Department of Anatomy, Yong Loo Lin School of Medicine, National University of Singapore, Singapore 117597, Singapore; 7 Department of Electron Microscope, Zhongshan School of Medicine, Sun Yat-sen University, Guangzhou, Guangdong 510080, China

## Abstract

This study attempted to graft neurotrophin-3 (NT-3) receptor (TrkC) gene modified mesenchymal stem cells (TrkC-MSCs) into the demyelinated spinal cord and to investigate whether electroacupuncture (EA) treatment could promote NT-3 secretion in the demyelinated spinal cord as well as further enhance grafted TrkC-MSCs to differentiate into oligodendrocytes, remyelination and functional recovery. Ethidium bromide (EB) was microinjected into the spinal cord of rats at T10 to establish a demyelinated model. Six groups of animals were prepared for the experiment: the sham, PBS, MSCs, MSCs+EA, TrkC-MSCs and TrkC-MSCs+EA groups. The results showed that TrkC-MSCs graft combined with EA treatment (TrkC-MSCs+EA group) significantly increased the number of OPCs and oligodendrocyte-like cells differentiated from MSCs. Immunoelectron microscopy showed that the oligodendrocyte-like cells differentiated from TrkC-MSCs formed myelin sheaths. Immunofluorescence histochemistry and Western blot analysis indicated that TrkC-MSCs+EA treatment could promote the myelin basic protein (MBP) expression and Kv1.2 arrangement trending towards the normal level. Furthermore, behavioural test and cortical motor evoked potentials detection demonstrated a significant functional recovery in the TrkC-MSCs+EA group. In conclusion, our results suggest that EA treatment can increase NT-3 expression, promote oligodendrocyte-like cell differentiation from TrkC-MSCs, remyelination and functional improvement of demyelinated spinal cord.

Demyelination occurs in several disorders in the central nervous system (CNS), including multiple sclerosis (MS) and spinal cord injury (SCI). Demyelination is an important cause of neurological deficits because it either delays or blocks impulse conduction^1^,^2^,^3^. Demyelinated axons can be repaired by remyelination in both humans^4^,^5^ and animals. Indeed, in some experimental models of demyelination repair can be, effectively complete, achieved either by endogenous Schwann cells^1^,^6^ or oligodendrocytes^7^,^8^. Moreover, remyelination has also been achieved by the transplantation of a variety of exogenous myelin-producing cells into experimentally demyelinated lesions. The role of therapeutic strategies based on cell replacement for demyelination diseases has been confirmed by numerous studies using myelin-producing cells, such as oligodendrocyte precursor cells (OPCs)^9^,^10^, Schwann cells^11^ or olfactory ensheathing cells^12^, and stem cells^9^.

Bone marrow mesenchymal stem cells (MSCs) are considered to be the most promising candidate in adult stem cell-based therapy for nervous system diseases because of their potential for easy collection, rapid proliferation, readily genetic manipulation, and their potential for clinical autograft. Moreover, there are a number of features that make MSCs attractive for cell implantation therapies in MS, including immunomodulation^13^, neuroprotection^14^ and cell-replacement^15^,^16^. Many studies have shown that MSCs implantation exerts a therapeutic effect in experimental autoimmune encephalomyelitis (EAE) or toxin-induced demyelinated models, which is supported by the evidences of functional repair and extensive remyelination^17^,^18^,^19^.

Electroacupuncture (EA) which originated in ancient China thousands of years ago is widely used as an adjuvant therapy for many diseases^20^,^21^,^22^,^23^,^24^, especially neurological diseases, including CNS damage and demyelinating diseases. EA has long been used to treat MS in traditional Chinese medicine, but the therapeutic mechanism is still unclear. There is evidence that EA can treat MS through modulating immune functions^24^. In this connection, EA on Governor Vessel (GV-EA) acupoints is commonly used to treat spinal cord injury because impairment of Governor Vessel is correlated with the damage of spinal cord in Chinese traditional medicine. Indeed, GV-EA has been shown to alleviate the secondary damage after spinal cord injury in animal models^21^,^22^,^25^. Our previous studies have reported that GV-EA could promote the secretion of neurotrophin-3 (NT-3) in injured spinal cord^22^,^26^,^27^. Other studies have also demonstrated that EA can increase the expression of some neurotrophic factors like NT-3, brain-derived neurotrophic factor (BDNF), nerve growth factor (NGF) and neurotrophin 4/5 (NT-4/5)^28^,^29^. NT-3 plays important roles in oligodendrocyte development^30^,^31^. It promotes the survival, proliferation and differentiation of OPCs, and myelination *in vitro* and *in vivo*^32^,^33^,^34^,^35^.

Our previous study indicated that EA treatment could promote NT-3 expression, increase the number and differentiation of endogenous OPCs, and remyelination in the demyelinated spinal cord^27^. However, the number of oligodendrocytes differentiated from the endogenous OPCs is limited. In addition, it is known that NT-3 promotes the survival and differentiation of cells by preferentially binding to its receptor TrkC. In this study, we attempted to graft TrkC gene modified MSCs (TrkC-MSCs) into the demyelinated spinal cord, to investigate whether EA treatment could promote NT-3 secretion in the demyelinated spinal cord, and further enhance grafted TrkC-MSCs to differentiate into oligodendrocytes, remyelination and functional recovery. We show here strong evidence that EA treatment can increase NT-3 level of demyelinated spinal cord and promote the differentiation of TrkC-MSCs into oligodendrocyte-like cells and remyelination as well as the nerve conduction functional improvement of demyelinated spinal cord.

## Results

### TrkC gene modified MSCs express stably TrkC protein in vitro and in vivo

**Figure 1:**
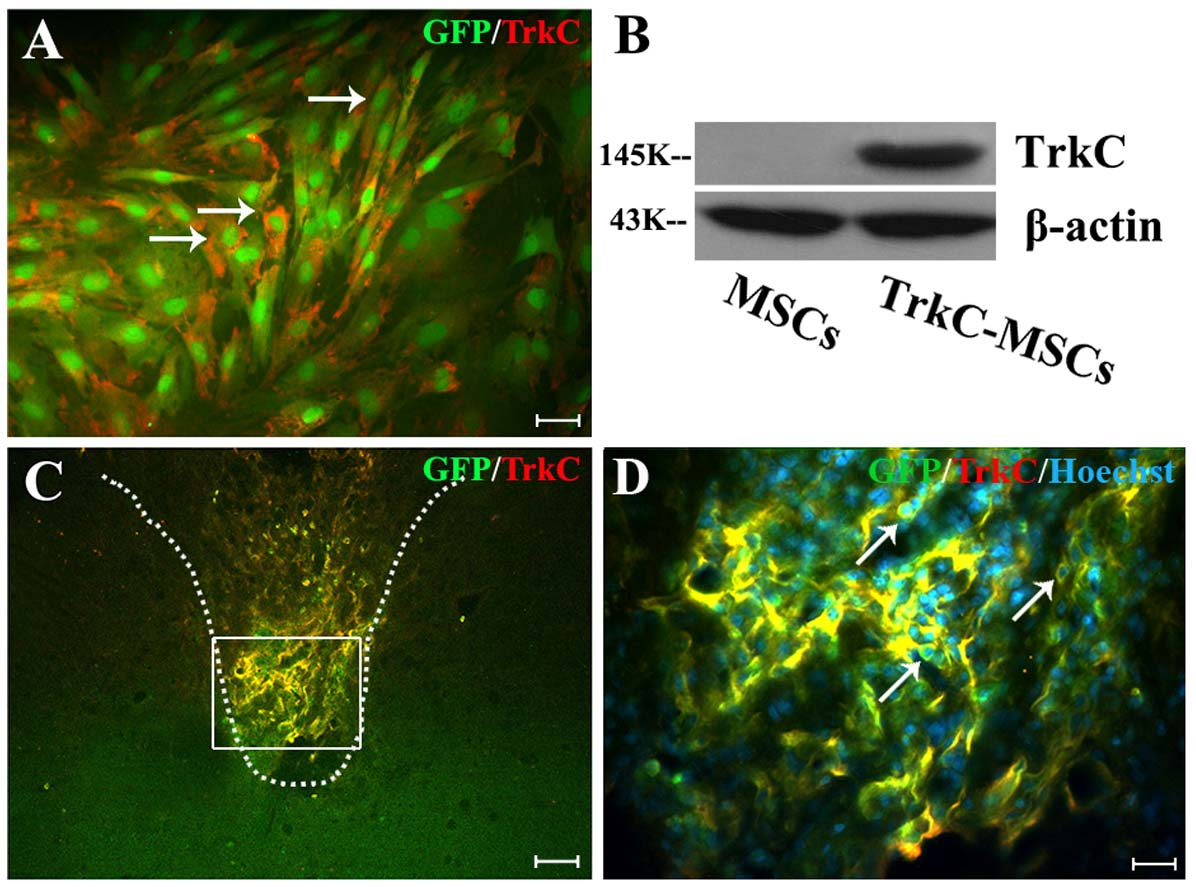
*In vitro* and *in vivo* analysis of adenoviral (Ad) vector-mediated transgene expression. (A) Performing TrkC immunofluorescence staining, 48 h after infection GFP-MSCs with Ad-TrkC. More than 80% cultured GFP-MSCs (green) expressed the TrkC gene product (red, arrows). Scale bar: 20 μm. (B) Transgenic MSCs were analyzed for the presence of TrkC using Western blot, 48 h after Ad vector transduction. Ad-TrkC transduced MSCs expressed TrkC protein, but TrkC protein could not be detected in non-transduced MSCs. Gels/blots were run under the same experimental conditions and β-actin was shown as a control. The cropped blots images were shown in the full-length blots are presented in Supplementary Figure 1. (C) *In vivo* confirmation of Ad vector-mediated TrkC expression in the GFP-MSCs (yellow) at 30 d after EB injection. (D) Showing higher magnification of GFP/TrkC/Hoechst33342 positive MSCs (yellow, arrows) in the rectangle boxes of (C). Scale bars: (C) = 80 μm; (D) = 20 μm.

MSCs cultures were efficiently transduced with adenoviral vectors encoding NT-3 receptor (TrkC) (MOI 300; Fig. 1A). High levels of transgene expression were seen *in vitro*; >80% of the cells expressed TrkC after infection of 2 days. TrkC overexpressing MSCs (TrkC-MSCs) displayed morphology similar to control MSCs cultures. Transgenic MSCs were analyzed for the presence of TrkC using Western blotting 2 days after adenoviral (Ad) vector transduction. Ad-TrkC transduced MSCs expressed TrkC protein, but TrkC protein could not be detected in non-transduced MSCs (Fig. 1B). *In vivo* analysis of transgene expression showed that a large number of TrkC-positive GFP-MSCs were detected within or nearby the demyelination/graft site of spinal cord in the TrkC-MSCs+EA group (Fig. 1C–D). Thus, the results indicate that Ad-TrkC transduced MSCs can express stably TrkC protein *in vitro* and *in vivo*.

### TrkC-MSCs graft & EA treatment increase NT-3 level in the demyelinated spinal cord

**Figure 2:**
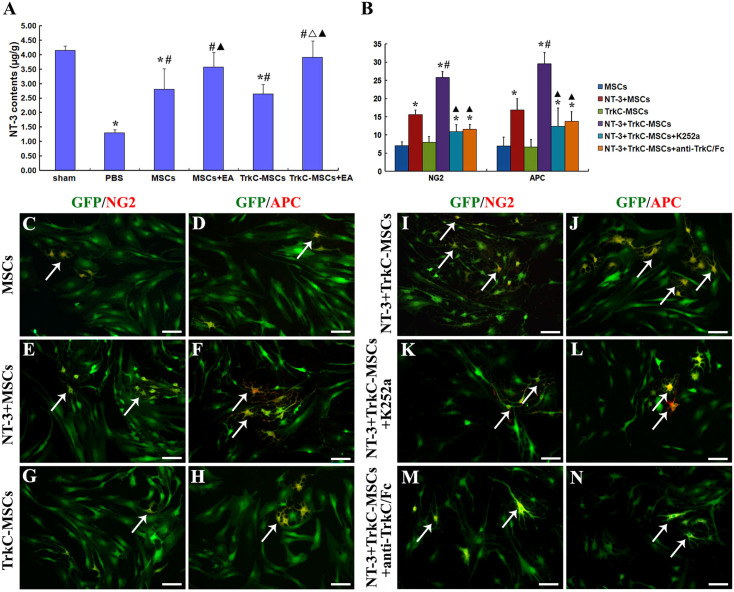
(A) NT-3 contents of the demyelinated spinal cords in six groups were measured by ELISA at 14 d after EB injection. Compared with the sham group, the NT-3 contents were significantly decreased in the PBS, MSCs, and TrkC-MSCs groups (* indicates p < 0.05). The NT-3 contents were significantly increased in the MSCs, MSCs+EA, TrkC-MSCs and TrkC-MSCs+EA groups as compared with the PBS group (^#^ indicates p < 0.05). The NT-3 content was significantly higher in the TrkC-MSCs+EA group than that in the MSCs group (^Δ^ indicates p < 0.05). The NT-3 contents were significantly higher in the MSCs+EA and TrkC-MSCs+EA groups than that in the TrkC-MSCs group (^▴^ indicates p < 0.05). Moreover, the NT-3 contents in the the MSCs+EA and TrkC-MSCs+EA groups had no significant difference as compared with the sham group (p > 0.05). Data = means ± SD. (B–N) Exogenous NT-3 promoting the differentiation of TrkC-MSCs into NG2 positive and APC positive oligodendrocyte-like cells. (B) Statistical analyses showed that the percentage of NG2 and APC positive oligodendrocyte-like cells was significantly increased in the NT-3+MSCs, NT-3+TrkC-MSCs, NT-3+TrkC-MSCs+K252a or NT-3+TrkC-MSCs+anti-TrkC/Fc groups as compared with the MSCs or TrkC-MSCs groups (* indicates p < 0.01). The percentage of NG2 and APC positive cells in the NT-3+TrkC-MSCs group was the highest and significantly higher than other five groups (^#^ indicates p < 0.01); In addition, the increase of NG2 and APC positive cells in the NT-3+TrkC-MSCs group was prevented by application of K252a or anti-TrkC/Fc antibody (the NT-3+TrkC-MSCs group *vs.* the NT-3+TrkC-MSCs+K252a or NT-3+TrkC-MSCs+anti-TrkC/Fc groups, ^▴^ indicates p < 0.01). (C–N) The immunocytochemistry showed that some green MSCs differentiated into NG2 and APC positive oligodendrocyte-like cells (yellow, arrows) in the MSCs (C and D), NT-3+MSCs (E and F), TrkC-MSCs (G and H), NT-3+TrkC-MSCs (I and J), NT-3+TrkC-MSCs+K252a (K and L) and NT-3+TrkC-MSCs+anti-TrkC/Fc (M and N) groups. Scale bar = 40 μm.

Two weeks following EB injection, the NT-3 concentration in the demyelinated spinal cord segments in six groups was measured by ELISA. The mean levels of the NT-3 content in three segments of injured spinal cord were considered in 6 groups and presented in Fig. 2A. As compared with the sham group, the NT-3 contents were significantly decreased in the PBS, MSCs, and TrkC-MSCs groups (p < 0.05). However, the NT-3 contents were significantly increased in the MSCs, MSCs+EA, TrkC-MSCs and TrkC-MSCs+EA groups as compared with the PBS group (p < 0.05). The NT-3 content was significantly higher in the TrkC-MSCs+EA group than that in the MSCs or TrkC-MSCs group (p < 0.05). Moreover, NT-3 concentration in the TrkC-MSCs+EA group was not significantly different from that of the sham group or MSCs+EA group (p > 0.05). The results indicate that grafted TrkC-MSCs combined with EA therapy can increase NT-3 level in the demyelinated spinal cord. These results are consistent with our previous results^22^,^27^. Moreover, our previous results showed that NT-3 can be produced by neurons, astrocytes, oligodendrocytes and microglia/macrophages in the transected spinal cord injury^22^ and the demyelinated spinal cord^27^.

### Exogenous NT-3 promotes differentiation of TrkC-MSCs into oligodendrocytes *in vitro*

In order to detect the effect of NT-3 on differentiation of TrkC-MSCs into oligodendrocytes, we added exogenous NT-3 into the cultured MSCs or TrkC-MSCs to ascertain whether NT-3 could promote the differentiation of TrkC-MSCs into oligodendrocytes *in vitro*. The cells were examined with NG2 and APC immunocytochemistry. The results demonstrated that MSCs differentiated into NG2 positive OPC-like cells and APC positive oligodendrocyte-like cells (yellow, Fig. 2C–N). The percentage of NG2 and APC positive cells was the lowest in the MSCs group (Fig. 2B–D). Exogenous NT-3 could moderately promote the differentiation of MSCs into NG2 and APC positive cells (Fig. 2B, E–F). The percentage of NG2 and APC positive cells in the NT-3+TrkC-MSCs group (Fig. 2B, I–J) was the highest and was significantly higher than the other groups (Fig. 2B). Moreover, the effect of NT-3 promoting the differentiation of TrkC-MSCs into oligodendrocyte-like cells was prevented by application of K252a (a specific inhibitor of neurotrophin-related tyrosine kinase) or anti-TrkC/Fc antibody in the NT-3+TrkC-MSCs+K252a (Fig. 2B, K–L) or NT-3+TrkC-MSCs+anti-TrkC/Fc (Fig. 2B, M–N) groups. These results indicated that NT-3 can promote the differentiation of TrkC-MSCs into NG2 positive OPC-like cells and APC positive oligodendrocyte-like cells by binding to its receptor TrkC.

### TrkC-MSCs graft & EA treatment improve behavioural outcome and cortical motor evoked potentials (CMEP)

**Figure 3:**
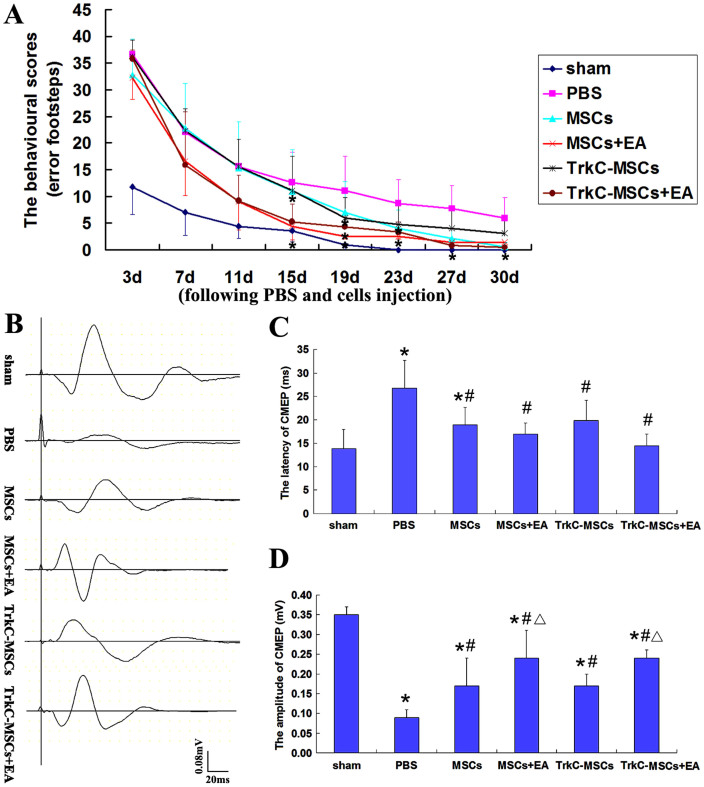
TrkC-MSCs graft & EA treatment improve behavioural outcome and cortical motor evoked potentials (CMEP). (A) Comparison of behavioural scores (error footsteps scores) among the sham, PBS, MSCs, MSCs+EA, TrkC-MSCs and TrkC-MSCs+EA groups. With increased time, all the rats showed a gradual improvement until the end of the experiment, but this effect was more pronounced in the TrkC-MSCs+EA and MSCs+EA groups. As compared with the PBS group, * indicates p < 0.05. Data = means ± SD. (B) The waveforms of cortical motor evoked potentials (CMEP) in six groups. (C) Statistical analysis of the latency of CMEP showed: As compared with the sham group, * indicates p < 0.05; As compared with the PBS group, ^#^ indicates p < 0.05. (D) Statistical analysis of the amplitude of CMEP showed: As compared with the sham group, * indicates p < 0.05; As compared with the PBS group, ^#^ indicates p < 0.05; As compared with the MSCs group or TrkC-MSCs groups, Δ indicates p < 0.05.

Within 3 days after EB injection, rats exhibited a staggering and retardant gait in a beam walking test. Considering the effect of injection procedure itself on the locomotion, rats in all the groups showed an immediately elevated error score even in the sham group. Rats receiving PBS injection showed many error footsteps and the highest error scores at all-time points (Fig. 3A). With increased time, all the rats showed a gradual improvement until the end of the experiment at 30 days following cells graft, but this effect was more pronounced in the MSCs+EA and TrkC-MSCs+EA group. After MSCs+EA and TrkC-MSCs+EA treatment, the behavioural function of rats were significantly improved compared with the PBS group beginning from the 15th day (p < 0.05, Fig. 3A). After TrkC-MSCs+EA and MSCs+EA treatment, the animal behaviour gradually recovered with advancing time so that normal function was restored by the end of the experiment at 30 days. The results suggest that TrkC-MSCs+EA and MSCs+EA treatment may effectively promote the functional recovery after the demyelinating injury.

To assess functional recovery following demyelination, CMEP responses were utilized as an *in vivo* functional measurement of axonal conduction in the DF of spinal cord. The latency and amplitude of CMEP were detected for each animal (Fig. 3B). The amplitude may be regarded to reflect the intensity of action potential which is determined by the normal common axonal quantity. The latency reflects the conductive velocity of action potential. As compared with the sham group, the latency and amplitude of CMEP in the PBS, MSCs and TrkC-MSCs groups was respectively prolonged and reduced (Fig. 3B–D). Compared with the PBS group, the latency of CMEP was significantly shorter and the amplitude was higher in the TrkC-MSCs+EA group and MSCs+EA group (Fig. 3B–D). Statistical analysis indicated that the CMEP in the TrkC-MSCs+EA group displayed higher amplitude than those in the MSCs or TrkC-MSCs groups (p < 0.05, Fig. 3D). However, there was no significant difference between the TrkC-MSCs+EA and MSCs+EA groups (p > 0.05, Fig. 3C–D). The results suggest that TrkC-MSCs+EA and MSCs+EA treatment may effectively promote the recovery of nerve conduction function after the demyelinated lesion.

### EA promotes grafted TrkC-MSCs to differentiate into oligodendrocyte-like cells

**Figure 4:**
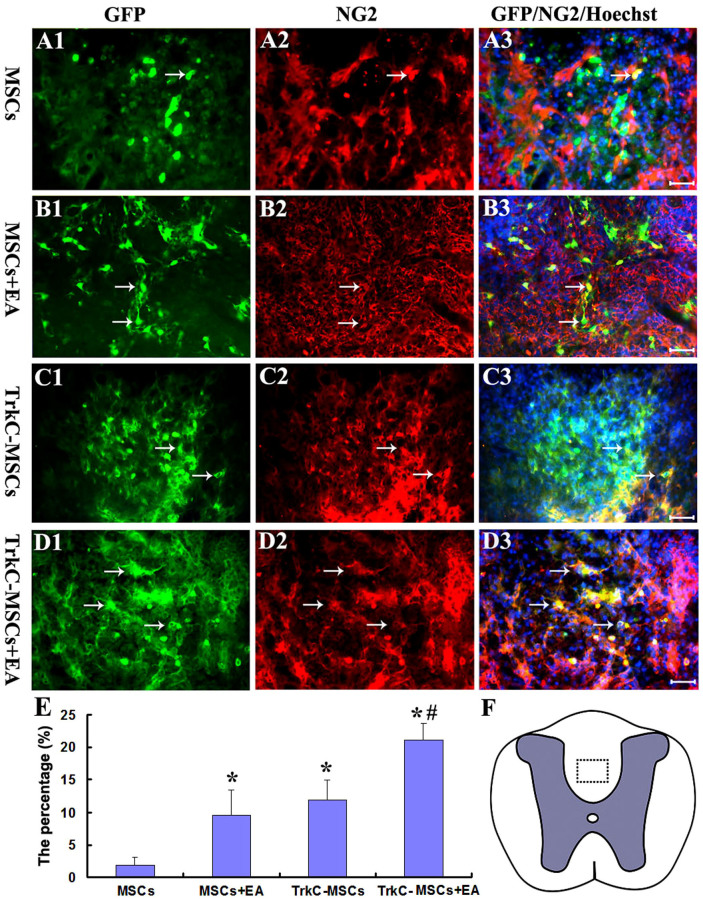
Differentiation of MSCs into NG2 positive young oligodendrocyte-like cells at 30 d following MSCs graft. GFP positive MSCs (green, arrows) and NG2 (red, arrows) double-labeling show the NG2/GFP positive oligodendrocyte-like cells (yellow, arrows) in the MSCs (A1–A3), MSCs+EA (B1–B3), TrkC-MSCs (C1–C3) and TrkC-MSCs+EA (D1–D3) groups. Scale bars = 20 μm. (E) Statistical analyses showed that the percentage of NG2/GFP positive cells differentiated from grafted cells in the TrkC-MSCs+EA group was higher than that in other three groups (the TrkC-MSCs+EA or MSCs+EA groups *vs.* the MSCs or TrkC-MSCs groups, * indicates p < 0.01; the TrkC-MSCs+EA group *vs.* the MSCs+EA group, ^#^ indicates p < 0.01). (F) The dotted box in a schematic diagram of spinal cord shows the figures site of (A1–D3) in the dorsal funiculus (DF).

The grafted GFP-MSCs transduced with Ad vectors encoding TrkC could easily be identified with fluorescence microscopy. Labeled MSCs were mainly found in the demyelination/graft site of DF of spinal cord and were well integrated with the host tissue (Fig. 1C–D). In a transverse section of the spinal cord processed for immunofluorescence labelling, the results showed that grafted GFP-MSCs differentiated into oligodendrocyte-like cells. NG2 is one of the markers for OPCs. Many NG2/GFP positive OPC-like cells were found in the demyelination/graft site of spinal cord in the MSCs (Fig. 4A1–A3), MSCs+EA (Fig. 4B1–B3), TrkC-MSCs (Fig. 4C1–C3) and TrkC-MSCs+EA (Fig. 4D1–D3) groups. In the TrkC-MSCs+EA group, the incidence of NG2/GFP positive OPC-like cells was the highest with some cells bearing long extending processes (Fig. 4D1–D3). Cell quantitative analysis showed the percentage of NG2/GFP positive OPC-like cells was highest in the TrkC-MSCs+EA group and lowest in the MSCs group (p < 0.01, Fig. 4E). Moreover, the percentage of NG2/GFP positive OPC-like cells in the TrkC-MSCs+EA group was higher than that in the MSCs+EA group (p < 0.01, Fig. 4E).

**Figure 5:**
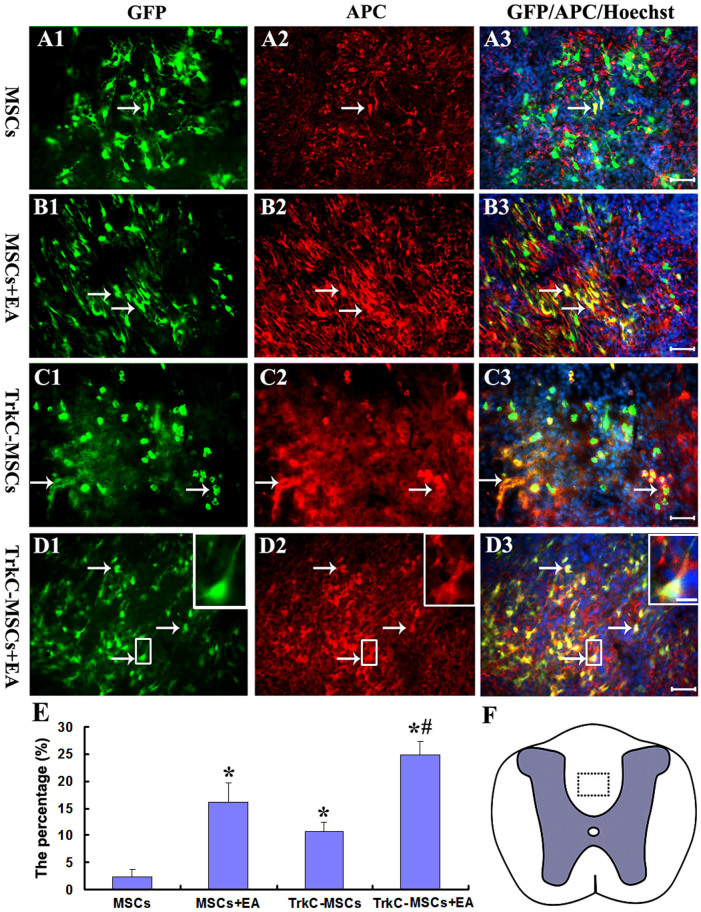
Differentiation of MSCs into APC positive mature oligodendrocyte-like cells at 30 d following MSCs graft. GFP positive MSCs (green, arrows) and APC (red, arrows) double-labeling show the APC/GFP positive oligodendrocyte-like cells (yellow, arrows) in the MSCs (A1–A3), MSCs+EA (B1–B3), TrkC-MSCs (C1–C3) and TrkC-MSCs+EA (D1–D3) groups. Scale bars = 20 μm. Insets show higher magnification images of the rectangle boxes in (D1–D3). (E) Statistical analyses showed that the percentage of APC/GFP positive cells differentiated from grafted cells in the TrkC-MSCs+EA group was the highest, and higher than that in other three groups (the TrkC-MSCs+EA, MSCs+EA or TrkC-MSCs groups *vs.* the MSCs group, * indicates p < 0.01; the TrkC-MSCs+EA group *vs.* the MSCs+EA or TrkC-MSCs groups, ^#^ indicates p < 0.01; the MSCs+EA group *vs.* the TrkC-MSCs group, p < 0.05). (F) The dotted box in a schematic diagram of spinal cord shows the figures site of (A1–D3) in the dorsal funiculus (DF).

In addition, we detected APC (a marker for mature oligodendrocyte) and GFP double labelled cells at the demyelination/graft site. The APC/GFP positive oligodendrocyte-like cells appeared irregular with some of them distributed in the demyelination sites of DF in the MSCs (Fig. 5A1–A3), MSCs+EA (Fig. 5B1–B3), TrkC-MSCs (Fig. 5C1–C3) and TrkC-MSCs+EA (Fig. 5D1–D3) groups. This feature was most pronounced in the TrkC-MSCs+EA group in which the majority of APC/GFP positive oligodendrocyte-like cells displayed long processes (insets in Fig. 5D1–D3). Cell quantitative analysis showed that the percentage of APC/GFP positive cells in the TrkC-MSCs+EA group was the highest among all groups (p < 0.01, Fig. 5E). A feature worthy of note was that the percentage of APC/GFP positive cells in the TrkC-MSCs+EA group was higher than that in the MSCs+EA group (p < 0.01, Fig. 5E). The percentage of APC/GFP positive cells in the TrkC-MSCs group was higher than that in the MSCs group. The results suggest that TrkC overexpression was beneficial to the differentiation of MSCs into oligodendrocyte-like cells.

**Figure 6:**
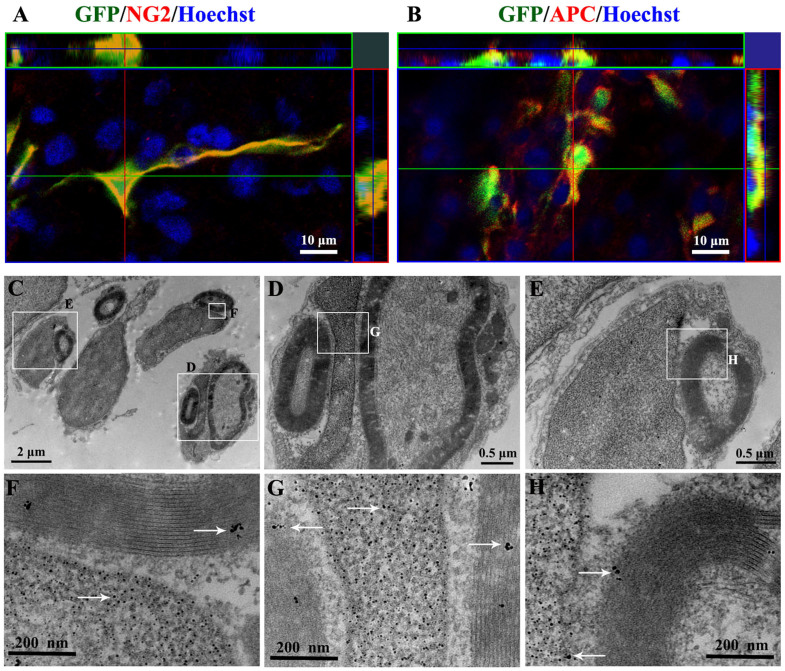
Confocal microscope and immunoelectron microscope images showed the differentiation of grafted MSCs into the oligodendrocyte-like cells within the demyelination site of spinal cord in the TrkC-MSCs+EA group. (A) The confocal imaging confirmed the colocalization of NG2 expression (red) and GFP positive MSCs (green) grafted. (B) The confocal imaging confirmed the colocalization of APC expression (red) and GFP positive MSCs (green) grafted. (C) Showing four GFP positive cells giving rise to the myelin profiles encircling an axon respectively. (D, E) Showing the higher magnification of the rectangle boxes in (C). Some GFP reaction products (gold-particles, white arrows) exist in the nucleus, myelin and cytoplasm of the grafted cells ((F, G and H) showing higher magnification of the rectangle boxes in (C–E)).

The confocal imaging showed the differentiation of GFP positive MSCs grafted into NG2 positive young oligodendrocyte-like cells (Fig. 6A) or APC positive oligodendrocyte-like cells (Fig. 6B) in the TrkC-MSCs+EA group. The three-dimensionally reconstructed immunofluorescence picture displayed the localization of NG2/GFP and NG2/GFP expression. To further investigate whether the grafted cells could differentiate into myelin-forming cells in the TrkC-MSCs+EA group, we performed immunoelectron microscopy using a GFP antibody. By this, the grafted GFP positive cells were identified with certainty within the demyelination/graft site and, furthermore, they were associated with myelin profiles encircling the axons within the demyelination/graft site (Fig. 6C–E). Some GFP reaction products labelled by high electron-dense gold-particles (Fig. 6F–H, white arrows) were precisely localized in the cytoplasm and nucleus of the grafted cell. Taken together, the morphological ultrastructural evidence suggests that grafted GFP positive cells can differentiate into myelin-forming cells.

### TrkC-MSCs graft & EA treatment promote new myelin forming

**Figure 7:**
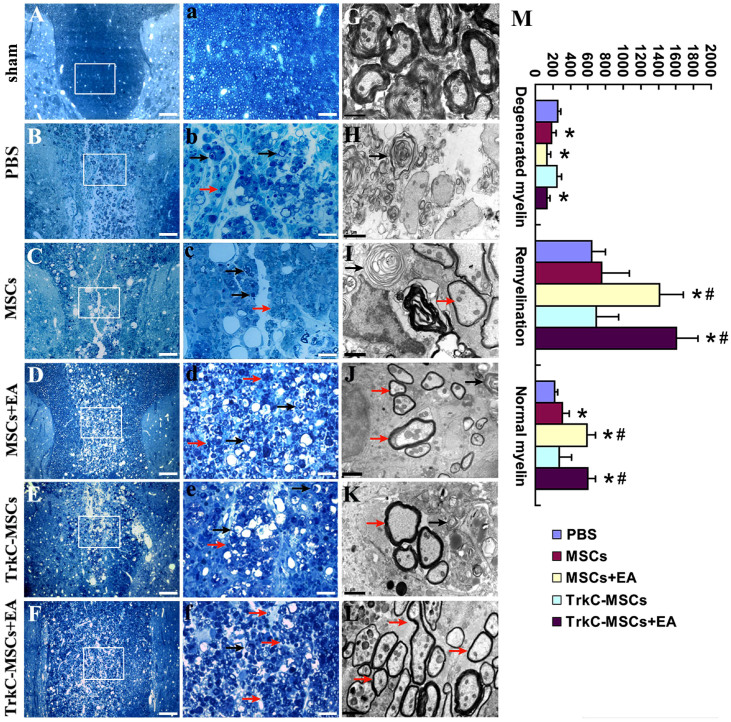
Myelin counting and electron microscopy. (A–F) Toluidine blue-stained semithin sections in the sham (A), PBS (B), MSCs (C), MSCs+EA (D), TrkC-MSCs (E) and TrkC-MSCs+EA (F) groups. (a–f) Showing higher magnification of the rectangle boxes in (A–F). Scale bars: (A–F) = 80 μm; (a–f) = 20 μm. Basically, many normal myelin sheaths are present in the sham (A and a). In the PBS group, the demyelination site was predominantly occupied by the demyelinated axons (black arrows) and debris of myelin sheaths when receiving EB injection only (B and b). Although MSCs (C and c) or TrkC-MSCs (E and e) transplantation also moderately increased the number of newborn myelin (remyelination, red arrows), considerable degenerated myelin (black arrows) still presented in the DF of spinal cord. It is notable that numerous remyelination (red arrows) were found in the MSCs+EA (D and d) and TrkC-MSCs+EA (F and f) groups. (G–L) Ultrastructural analysis in the sham (G), PBS (H), MSCs (I), MSCs+EA (J), TrkC-MSCs (K) and TrkC-MSCs+EA (L) groups showed the degenerated myelin (black arrows, exhibiting onion-like appearance whose myelin lamellae were disorganized and loosened.) and newborn myelin (red arrows, showing thinner thickness of myelin sheaths) in the demyelination/graft site of spinal cord. (M) Comparation of the number of three kinds of myelin (degenerated myelin, newborn myelin and normal myelin) among five groups (compared with the PBS group, * indicates p < 0.01; compared with the MSCs or TrkC-MSCs groups, ^#^ indicates p < 0.05).

In order to elucidate whether TrkC-MSCs graft combined with EA treatment could promote remyelination, semithin section and ultrastructural analysis were performed. We used toluidine blue-stained semithin sections of demyelinated spinal cord and classified three kinds of myelin sheath at 30 days: degenerated myelin (loose, redundant sheaths that form loops), newborn myelin (remyelination; thinner sheaths and lighter in color) and normal myelin (compact, thick dark sheaths). There were numerous normal myelin sheaths in DF of spinal cord in the sham group (Fig. 7A,a). In contrast, the EB-induced injury site was predominantly occupied by the demyelinated axons and macrophages when receiving PBS injection only (Fig. 7B,b). Although MSCs (Fig. 7C,c) or TrkC-MSCs (Fig. 7E,e) transplantation also moderately increased the number of newly formed myelin sheaths, considerable degenerated myelin sheaths and macrophages still presented in the DF of spinal cord. It is notable that numerous newborn myelin sheaths were found in the MSCs+EA (Fig. 7D,d) and TrkC-MSCs+EA (Fig. 7F,f) groups. These newborn myelin sheaths were thinner than those in the sham group. Quantitative analysis of three kinds of myelin among five groups showed that the number of normal myelin or newborn myelin in the MSCs+EA and TrkC-MSCs+EA groups was higher than that in the PBS, MSCs or TrkC-MSCs groups (p < 0.05, Fig. 7M).

By electron microscopy, many normal, thick and compact myelins were found in the sham group (Fig. 7G). In the PBS group, numerous degenerated myelin sheaths and swollen axons denuded of myelin sheath were observed in the demyelinated DF of spinal cord (Fig. 7H). The degenerated myelin sheaths exhibited onion-like appearance whose myelin lamellae were disorganized and loosened (Fig. 7H). In contrast, the less degenerated myelin sheaths and newborn myelin sheaths were common in the MSCs (Fig. 7I) or TrkC-MSCs (Fig. 7K) groups as compared with the PBS group (Fig. 7H). Very strikingly, numerous newborn myelin sheaths were found in the MSCs+EA (Fig. 7J) and TrkC-MSCs+EA (Fig. 7L) groups. In addition, ultrastructural analysis showed the presence of Schwann cell forming myelin sheaths (not shown) in the demyelination/graft site. Schwann cells forming myelin sheaths are identified by the presence of a nucleus adjacent to the myelin and the outer basement membrane. This feature is consistent with our previous observation^22^,^27^.

### TrkC-MSCs graft & EA treatment promote MBP expression and Kv1.2 arrangement trending to normal level

**Figure 8:**
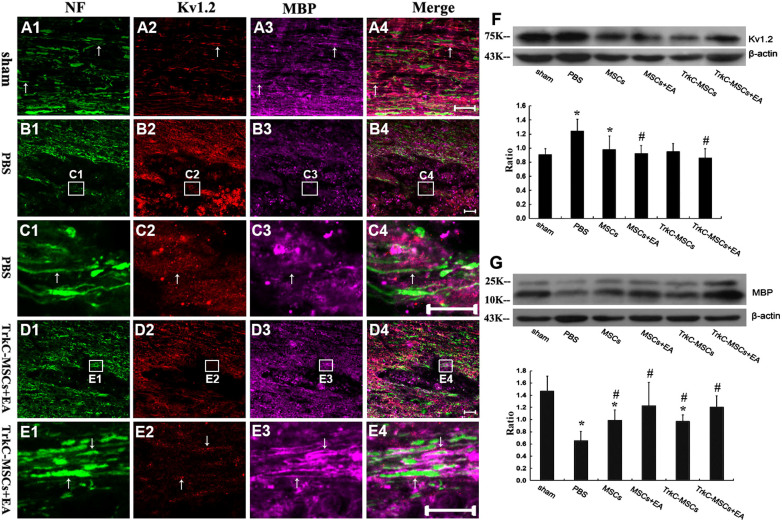
TrkC-MSCs graft & EA treatment promote MBP expression and Kv1.2 arrangement trending to normal level. NF (green, marker for axons)/Kv1.2 (red, marker for K^+^ channels)/MBP (purple, marker for myelin sheaths) triple-label immunofluorescence staining was performed to examine the expression and distribution of Kv1.2 and MBP in the longitude section containing the demyelination/graft site of spinal cord. (A1–A4) Showing the highly clustered pattern of Kv1.2 staining were observed in the DF of spinal cord, and Kv1.2 staining was clustered in the juxtaparanodal (JXP)-like regions under the MBP^+^ myelin sheath along myelinated axons in the sham group. (B1–B4) In the PBS group, the clustered pattern of Kv1.2 staining was decreased, and the distribution pattern of Kv1.2 staining was diffuse. In some demyelination sites, Kv1.2 staining appeared slightly increased, but not in clusters (B1). (C1–C4) Showing higher magnification of the rectangle boxes in (B1–B4). (D1–D4) In the TrkC-MSCs+EA group, Kv1.2 clusters were still present, but not very typical, most likely resulting from partial remyelination and/or demyelination, which were indicated by the reduction but not complete absence of the MBP positive myelin staining (D1–D4). Overall, disruption of Kv1.2 clustering was highly restricted to the demyelination/graft site. Scale bars = 20 μm. In non-demyelination areas, Kv1.2 clustering remained normal. (E1–E4) Showing higher magnification of the rectangle boxes in (D1–D4). (F–G) Western blot analysis of Kv1.2 and MBP expression in the sham, PBS, MSCs, MSCs+EA, TrkC-MSCs and TrkC-MSCs+EA groups. Gels/blots were run under the same experimental conditions and β-actin was shown as a control. The cropped gels/blots images were shown in the full-length gels/blots are presented in Supplementary Figure 2. Quantification of protein expression showed: As compared with the sham group, * indicates p < 0.05; As compared with the PBS group, ^#^ indicates p < 0.05.

NF (marker for axons)/Kv1.2 (a marker for K^+^ channels)/MBP (a marker for myelins) triple-label immunofluorescence staining was performed to examine the expression and distribution of Kv1.2 and MBP in the demyelination/graft site. In the sham group, the highly clustered pattern of Kv1.2 staining was observed in the DF of spinal cord (Fig. 8A1), and Kv1.2 staining was clustered in the juxtaparanodal (JXP)-like regions under the myelin sheath along myelinated axons (Fig. 8A1–A4). The clustered pattern of Kv1.2 staining was decreased or disappeared in the demyelination sites after EB injection, and the distribution pattern of Kv1.2 staining was diffuse. In some injury sites, Kv1.2 staining appeared slightly increased, but not in clusters (Fig. 8B1). The smooth staining signals were likely contributed by Kv1.2 expression in proliferating immune cells and demyelinated axons (Fig. 8B1–B4, C1–C4). In some injury sites in the TrkC-MSCs+EA group, Kv1.2 clusters were still present but atypical in shape, most likely resulting from partial remyelination and/or demyelination, as indicated by the reduction but not complete absence of the MBP positive myelin staining (Fig. 8D1–D4,E1–E4). Overall, disruption of Kv1.2 clustering was highly restricted to the injury site. In non-injury areas, Kv1.2 clustering remained normal.

Western blot analysis showed that the levels of Kv1.2 protein were increased in PBS and MSCs groups as compared with the sham group (*p < 0.05, Fig. 8F). As compared with the PBS group, Kv1.2 protein expression was decreased in the MSCs+EA and TrkC-MSCs+EA groups (#p < 0.05, Fig. 8F). Conversely, the expression levels of MBP protein were decreased in the PBS, MSCs and TrkC-MSCs groups as compared with the sham group (*p < 0.05, Fig. 8G). As compared with the PBS group, MBP protein expression was increased in the MSCs+EA and TrkC-MSCs+EA group (#p < 0.05, Fig. 8G).

## Discussion

We reported previously that EA treatment can promote NT-3 expression, and increase the number and differentiation of endogenous OPCs and remyelination in the demyelinated spinal cord^27^. However, the number of oligodendrocytes differentiated from the endogenous OPCs is limited. In addition, it is known that NT-3 promotes the survival and differentiation of cells by preferentially binding to its receptor TrkC. Therefore, in this study, we attempted to graft TrkC (NT-3 receptor) gene modified MSCs (TrkC-MSCs) into the demyelinated spinal cord, to investigate whether EA combined with TrkC-MSCs graft treatment could promote NT-3 secretion in the demyelinated spinal cord, and further enhance the grafted TrkC-MSCs to differentiate into oligodendrocytes, remyelination and functional recovery in the demyelinated spinal cord. Indeed, the present results suggest that EA combined with cells graft treatment could increase NT-3 level of demyelinated spinal cord, and promote oligodendrocyte-like cells differentiation from TrkC-MSCs and remyelination as well as the nerve conduction functional improvement in the demyelinated spinal cord.

Multiple sclerosis (MS) animal models include EAE, focal myelin toxin injection and cuprizone ingestion^36^,^37^. EAE exhibits characteristic features similar to many pathophysiological changes of MS, including demyelinating antibody attacking axons and increasing encephalitogenic T cells^38^,^39^,^40^. However, it has some disadvantages for experimental studies in view of the unpredictability of demyelination region and the variability of pathology as the disease progresses. Hence, it would be difficult to assess the effect of EA treatment on NT-3 level and differentiation of grafted MSCs. In view of the above, we have opted focal myelin toxin--EB injection into the white matter regions including the dorsal funiculus to kill oligodendrocytes and astrocytes. This produces a focal area of demyelination with accurate positioning and gradually results in lesion pathology^41^. It is of important significance that it can separate demyelination from remyelination, a spontaneous regenerative response subsequent to demyelination^42^,^43^. Aside from the demyelination, EB injection also induces a glia-free environment at its injected white matter of spinal cord, which was confirmed by our previous results of luxol fast blue (LFB) and immunofluoresence staining^27^. We have since successfully established the demyelinated model induced by EB.

Our earlier work showed that EA could increase NT-3 levels and promote the differentiation of endogenous OPCs and remyelination in the demyelinated spinal cord induced by EB^27^. In the present study, we attempted to apply TrkC-MSCs graft combined with EA treatment to further enhance the efficacy of remyelination in the demyelinated dorsal funiculus of spinal cord. Consequently, we found that the EA significantly promoted the differentiation of TrkC-MSCs into oligodendrocyte-like cells, which was accompanied by elevation of NT-3 level. Very interestingly, the EA effect alone could increase the NT-3 level (1.94 ± 0.22 μg/g) as compared with the EB (1.10 ± 0.14 μg/g) group^27^. However, the NT-3 level of the demyelinated spinal cord in the EA+MSCs (3.58 ± 0.51 μg/g) or EA+TrkC-MSCs (3.91 ± 0.58 μg/g) groups was further elevated as compared with EA treatment alone. In our previous study, we showed that some grafted MSCs were NT-3 immunopositive cells in the MSCs+EA group^27^. We consider the increased NT-3 content in the MSCs+EA or TrkC-MSCs+EA groups was attributed to a synergistic effect of EA treatment and MSCs transplantation. This takes into consideration of reports that transplanted MSCs can produce NT-3^44^ or stimulate neuroglial cells to produce neurotrophic factors^45^ in the CNS.

The present results suggest that EA can promote the survival and differentiation of grafted TrkC-MSCs by increasing NT-3 levels in the demyelinated spinal cord. NT-3 is a significant member of the neurotrophic factors family and plays an important role in regulating the normal oligodendrocyte development and the quantity of oligodendrocytes and myelin regeneration following CNS injury and demyelination^46^,^47^. The TrkC is the high-affinity receptor of NT-3. *In vivo* studies utilizing NT-3 and TrkC knockout mice have provided valuable information with respect to the importance of both ligand and receptor during development of peripheral nervous system and CNS^48^,^49^,^50^. In the present study, in order to examine the effect of NT-3 on the differentiation of TrkC-MSCs, we added the exogenous NT-3 into the cultured MSCs or TrkC-MSCs to observe whether NT-3 could promote the differentiation of TrkC-MSCs into oligodendrocytes *in vitro*. Our results indicate that NT-3 can promote differentiation of TrkC-MSCs into oligodendrocyte-like cells. Moreover, the efficiency of NT-3 promoting the differentiation of TrkC-MSCs was prevented by application of K252a (a specific inhibitor of neurotrophin-related tyrosine kinase) and anti-TrkC-Fc IgG. In addition, the immunoelectron microscopy further demonstrated that the EA could promote grafted TrkC-MSCs to differentiate into oligodendrocyte-like cells which appeared to enwrap axons forming new myelins in the demyelination/graft site of spinal cord. The aforementioned studies have demonstrated that NT-3 binding to its high-affinity receptor TrkC promotes the differentiation of TrkC-MSCs into myelin-forming cells. The underlying mechanism is not fully clear so far. NT-3 does so via two distinct and separable signaling pathways: the PI3-kinase and MEK pathways [mitogen-activated protein kinase (MAPK), extracellular signal-regulated protein kinase 2 (ERK2)], which are common downstream substrates of TrkC receptor tyrosine kinases^33^. Studies have also reported that NT-3 can induce both the survival and proliferation of OPCs and differentiation of oligodendrocytes involving the transcription factor cAMP response element-binding protein (CREB)^46^,^47^.

Interestingly, in this study, we found that EA could promote grafted TrkC-MSCs differentiated only into oligodendrocyte-like cells, but not into neurons and astroglia cells (not shown) in the demyelination/graft site of spinal cord. However, our previous study showed that EA could promote grafted TrkC-MSCs to differentiate into neuron-like and oligodendrocyte-like cells in the completely transected spinal cord^22^. Comparison of these results suggests that the differentiation of MSCs overexpressing TrkC may also be regulated by the local microenvironment. The dorsal funiculus of spinal cord is mainly composed of myelinated nerve fibers and oligodendrocytes. It is speculated that the local microenvironment of the demyelinated region may be more conducive to induce the differentiation of grafted stem cells into oligodendrocytes.

Myelin sheath is critical for the normal functioning of the vertebrate nervous system. In the CNS, myelin sheaths are produced by oligodendrocytes. In the present study, the semithin sections and ultrastructural analysis showed that there were more newly formed myelin sheaths in the TrkC-MSCs+EA group, and Western blot analysis further demonstrated that the levels of MBP protein were increased in the TrkC-MSCs+EA group. In the latter, the following are possible explanations for the efficient remyelination. Firstly, grafted TrkC-MSCs differentiate into oligodendrocytes forming new myelin as evidenced by immunohistochemistry and immunoelectron microscopy. Secondly, the involvement of local endogenous OPCs in the demyelination/graft site should be considered during remyelination. Our studies along with others have demonstrated that EA treatment can increase the tissue cAMP level and the expression of neurotrophic factors, such as NT-3, BDNF and GDNF^20^,^29^,^51^, which can promote OPCs differentiation into mature oligdendrocytes to form myelin. We reported previously that EA could promote proliferation and differentiation of endogenous OPCs, and remyelination in the demyelinated spinal cord injury induced by EB^27^. Thus, it is suggested that EA can mobilize endogenous OPCs to repair the damaged nervous system at least to a certain extent. MSCs may also activate endogenous progenitor cells and facilitate endogenous CNS repair^52^,^53^,^54^. As a corollary, we postulate that TrkC-MSCs combined with EA treatment could also activate endogenous myelin repair. Thirdly, it is well established that many demyelinated axons are acutely remyelinated by Schwann cells in the spinal cord demyelinating lesion induced by EB injection into the dorsal columns^1^. By electron microscopy, we had observed that some Schwann cells formed myelin sheaths in the demyeliation site of spinal cord.

We have observed many more newborn myelin sheaths accompanied by a better behavioural recovery and electrophysiological improvement in the TrkC-MSCs+EA group. This suggests that TrkC-MSCs+EA treatment can promote functional recovery by increasing remyelination. Myelination of axons allows a fast saltatory conduction of electrical impulses, and provides support both mechanically and functionally by cellular communication between the axon and the oligodendrocyte which produces the myelin sheath. Demyelination of axons reduces the conduction velocity of nerve impulses, but also makes the axons vulnerable to degeneration^42^. Therefore, the loss of oligodendrocytes and myelin sheaths would result in severe functional impairment. Although spontaneous remyelination occurs in chronic demyelinating diseases, such as MS, the repair process eventually fails, often resulting in long-term disability^55^. Studies also demonstrated that remyelination will lead to restoration of function in clinic MS and experimental models of demyelination^3^,^56^,^57^. Thus, the above studies have suggested that the extensive remyelination would form the structural basis for the electrophysiological and behavioural recovery.

Myelination is a complex process which results in a precise spatial localization of ion channels on the axonal membrane to ensure rapid and efficient axonal conduction. The electrophysiological and immunohistological studies have revealed that in adult mammalian axons Na^+^ channels are localized at the node of Ranvier, while K^+^ channels are covered by myelin sheaths and are restricted to the juxtaparanodal (JXP) region^58^,^59^. It has been shown that the distribution of Na^+^ and K^+^ channels is altered in the animal models of dysmyelinated spinal cord^1^,^60^,^61^ and demyelinating diseases including MS and EAE^58^,^62^. The ion channels are implicated in axon conduction failure and axonal degeneration^62^,^63^,^64^. The axonal K^+^ channels that hyperpolarize membrane potentials toward the resting level, play critical roles in regulating the initiation, waveform, frequency and uni-directional propagation of action potentials^65^,^66^. Normally, K^+^ channel subunits Kv1.1 and Kv1.2 are clustered in the JXP regions under the myelin sheath along myelinated axons in the brain and spinal cord^67^,^68^. The demyelinated axons exhibit a dispersed distribution of K^+^ channel subunits Kv1.1 and Kv1.2 with loss of the characteristic distinction of the juxtaparanodal and paranodal areas^69^,^70^. Moreover, a recent study has indicated that adult neural precursor cells (aNPCs) transplantation can successfully form compact myelin, reconstruct nodes of Ranvier and reverse long-term molecular abnormalities of Kv1.2 and Caspr in the adult dysmyelinated CNS axons^60^. In the present study, the results showed that TrkC-MSCs combined with EA treatment could promote myelination by oligodendrocytes differentiated from grafted cells and endogenous OPCs, and ion channel proteins reorganization into near normal spatial localization along the remyelinated axons. Therefore, our study indicates that TrkC-MSCs coupled with the EA treatment can promote remyelination and redistribution of clustered pattern of Kv1.2 to the JXP regions along the myelinated axons, which is the basic structure of axonal conduction recovery.

In conclusion, EA treatment can promote NT-3 expression in the demyelinated spinal cord, and further enhance the grafted TrkC-MSCs to differentiate into oligodendrocyte-like cells, remyelination as well as ion channel proteins reorganization into near normal spatial localization along remyelinated axons. It is conceivable that this would ultimately improve the nerve conduction and behavioural function of the demyelinated spinal cord.

## Methods

### MSCs preparation

The methods for the preparation of bone marrow mesenchymal stem cells (MSCs) have been described in detail in our previous study^22^. Briefly, the tibias and femurs of the green fluorescent protein (GFP) transgenic Sprague-Dawley (SD) rats (male, 2 weeks old, Osaka University, Osaka, Japan) were dissected under the anesthesia and aseptic conditions. After removing the ends of the bone, a 22-gauge needle filled with low glucose Dulbecco's Modified Eagle's Medium (L-DMEM, Gibco/BRL, Carlsbad, CA, USA) was injected into the central canal of the bone to extrude the bone marrow. The solution containing bone marrow was then centrifuged at 1000 rpm for 5 min. The pellet was resuspended in L-DMEM and supplemented with 10% inactivated fetal bovine serum (FBS), penicillin (100 U/ml), and streptomycin (100 mg/ml). The cells were then cultured in a 75-ml cell flask. After planting the cells for 48 h, the medium was replaced to remove non-adherent cells. When the adherent MSCs grew to near 70–80% confluency, they were serially passaged using 0.25% trypsin/0.02% EDTA. After being passaged 3–5 times, the MSCs were ready for use in transplantation.

### Transduction of MSCs with TrkC gene

Recombinant adenoviral vectors of human tyrosine kinase C (TrkC) gene (Ad-TrkC) were constructed as described in our previous study^22^. The recombinant adenovirus was propagated in 293 cells and concentrated on cesium chloride gradients after standard procedures (according to the BD Adeno-XTM instruction manual). The titer of the concentrated Ad-TrkC stock, determined by direct plaque assay, was 2.5 × 10^11^ plaque forming units (pfu)/ml.

MSCs were infected with Ad-TrkC at a multiplicity of infection (MOI) of 300 for 48 h as reported in our previous study^22^. After 48 h, the TrkC gene modified MSCs (TrkC-MSCs) were collected and were ready for use in transplantation. In addition, a small part of TrkC-MSCs were fixed in 2.5% paraformaldehyde for histochemical staining. The expression of TrkC was examined by immunofluorescent method. The primary antibody was mouse anti-human TrkC (1:500, R&D), and the secondary antibody was Cy3-conjugated goat anti-mouse IgG.

### A statement identifying the institutional and/or licensing committee experimental approval

All animal experiments were approved by the Institutional Animal Care and use Committee of Sun Yat-sen University and performed in accordance with the guidelines.

### Experimental animals, surgery and cells graft

A total of 111 Sprague-Dawley male (SD) rats (of body weight 250–300 g) were used in this study (Supplementary Table 1). The rats were housed in a temperature-controlled (24 ± 2°C) and light-controlled (12:12 light-dark cycle) room with free access to food and water. Prior to experimental manipulation, rats were allowed to acclimatize to the housing facilities and were handled daily at least for 3 days. All animals in the experiment were provided by Experimental Animal Center of Sun Yat-sen University and received humane care in compliance with the Public Health Service Guide for the Care and Use of Laboratory Animals. All efforts were made to minimize the number of animals used and their suffering.

The rats were anesthetized with an intraperitoneal (i.p.) injection of 1% sodium pentobarbital (40 mg/kg). Dorsal laminectomies were performed at the T9–T10 vertebral level and the dura was sheared open. One microliter of EB (0.1 mg/ml) was stereotaxically injected into 2 sites (at intervals of about 5 mm, 0.5 μl/site) at the central line of the T10 spinal dorsal funiculus (DF, at depths of 0.8 and 0.6 mm) using a 1 μl fixed-needle Hamilton syringe^7^. In the sham groups, laminectomies and exposure of the dura were carried out without EB injection. After surgery, all animals received an intramuscular injection of penicillin (160,000 U/ml/d) and then caged separately on thick soft bedding.

Before transplantation, 10 μg/ml Hoechst 33342 (Sigma) was used to label MSCs in the different treatment groups for 2 h. At the 4th day after EB injection, a 1 μl volume of MSCs suspension (1 × 10^5^/μl PBS) at an infusion rate of 0.5 μl/min was directly injected into the EB lesion site according to our previous study and by others^19^,^71^. However, in the PBS group, 1 μl PBS was injected instead of cell suspension (Supplementary Figure 3).

The rats were randomly divided into six groups (*n* = 18 for each group, total 108 rats): (1) the sham group, received dorsal laminectomy and exposure of the dura only; (2) the PBS group, received PBS injection at the 4th day after EB injection; (3) the MSCs group, received MSCs graft at the 4th day after EB injection; (4) the MSCs+EA group, received MSCs graft plus EA treatment at the 4th day after EB injection; (5) the TrkC-MSCs group, received TrkC-MSCs graft at the 4th day after EB injection; (6) the TrkC-MSCs+EA group, received TrkC-MSCs graft plus EA treatment at the 4th day after EB injection.

### Electro-acupuncture (EA) therapy

Three days after EB injection, rats were anesthetized as described above, and the T10 thoracic cord was exposed again for PBS or cells injection (Supplementary Figure 3). In the MSCs+EA and TrkC-MSCs+EA groups, rats received EA treatment every other day, beginning at the day following cells graft for 4 weeks. EA stimulation was performed at two acupoints in Governor Vessel. Two “Governor Vessel” acupoints were utilized during EA treatment, which are Jizhong (GV6) and Zhiyang (GV9)^27^. The acupoint GV6 is located on the posterior midline and in the depression below the spinous process of the eleventh thoracic vertebra in prone position. GV9 is located on the posterior midline and in the depression below the spinous process of the seventh thoracic vertebra in prone position, respectively. EA treatment on both GV6 and GV9 acupoints can directly treat the demyelinated spinal cord. Needles were connected with the output terminals of an electro-acupuncture apparatus (Model G 6805-2, Shanghai Medical Electronic Apparatus Company, China). Alternating strings of dense-sparse frequencies (60 Hz for 1.05 s and 2 Hz for 2.85 s alternately) were used for EA. The intensity was adjusted to induce slight twitch of the hindlimbs (≤1 mA), with the intensity lasting for 20 min. EA was administered every other day for 26 days, starting from the fourth day post-surgery.

### Training and behavioural scoring

Prior to EB injection, all animals were trained until they would cross an elevated 2 m-long, 15 mm-diameter wooden beam without stalling. After PBS and cells injection, all animals underwent behavioural testing and were videotaped every 3 days for 30 days (Supplementary Figure 3). The placement of footsteps made by each rat during two traverses of the beam in each direction was given a score of 0, 1 or 2. A score of 0 was given to each normal step, whereas a minor error (slight insecurity of foot placement) was scored 1 and a major error (foot slipped completely from beam surface) was scored 2^36^,^56^. Throughout the experiment, both observers were unaware of which rats had received cells graft or/and EA treatment and which had not.

### NT-3 detected by enzyme-linked immunosorbent assay (ELISA)

We reported previously that NT-3 level at 2 weeks after the spinal cord transection was increased after EA treatment^27^. In the present study, we investigated whether cells graft and EA treatment could induce a higher level of NT-3 in the demyelinated spinal cord. Fifteen days after cells graft and EA treatment, 30 rats of three groups (*n* = 5 for each group) were anesthetized with 1% sodium pentobarbital (40 mg/kg, i.p.) and transcardially perfused with 200 ml of ice-cold 0.1 M PB. The demyelinated spinal cord segment (about 0.5 mm) was excised while the cord was placed on dry ice. The segments were weighed and then mechanically homogenized in ice-cold 0.1 M PB. Homogenates were centrifuged for 10 min at 14,000 rpm at 4°C and used for NT-3 ELISA according to the instructions of the manufacturer (NT-3 Emax Immuno-Assay System, Boster, China).

### Effect of NT-3 on differentiation of TrkC gene modified MSCs *in vitro*

In order to examine the effect of NT-3 on the differentiation rate of oligodendrocyte-like cells from TrkC-MSCs, gene-modified cells were prepared first as described above. MSCs were transfetcted with Ad-TrkC (MOI = 300), and then both MSCs and TrkC-MSCs were adjusted to 2 × 10^4^/ml and cultured in a 24-well plastic plate in L-DMEM containing 5% FBS (~4000 cells/200 μl/per well). Six experimental groups were divided for each phenotypic verification: the MSCs, NT-3+MSCs, TrkC-MSCs, NT-3+TrkC-MSCs, NT-3+TrkC-MSCs+anti-TrkC/Fc and NT-3+TrkC-MSCs + K252a groups (4 wells/group, including 1 negative control well). In the NT-3+MSCs, NT-3+TrkC-MSCs, NT-3+TrkC-MSCs+anti-TrkC/Fc and NT-3+TrkC-MSCs+ K252a groups, the culture medium contained 20 ng/ml NT-3. In addition, 100 nM K252a (Calbiochem, Darmstadt, Germany), an inhibitor of neurotrophin-related tyrosine kinase, was added into the culture medium in the NT-3+TrkC-MSCs+K252a group. In the NT-3+TrkC-MSCs+anti-TrkC/Fc group, 0.2 μg/ml anti-human TrkC/Fc antibody (R&D, USA) was added into the culture medium. The culture medium was replaced every two days. After 7 days of culture, the cells were fixed and then immunostained with rabbit anti-a chondroitin sulfate proteoglycan (neuroglycan 2 (NG2), a marker for oligodendrocyte progenitor cells, 1:200, Millipore, USA) and rabbit anti-adenomatous polyposis coli (APC, a marker for mature oligodendrocytes, 1:1000, Calbiochem) overnight at 4°C, followed by incubation with the corresponding Cy3-conjugated secondary antibodies (1:800, Jackson ImmunoResearch, USA) for 1 h before being rinsed three times with 0.01 M PBS (pH 7.4). GFP cells (green), Cy3 immunofluorochemically labeled cells (red, for APC), and double-labeled cells (yellow) were evaluated via a fluorescence microscope (Leica DMIRB). For each culture well, four random microscopic visual fields at 400× magnification were observed. A total of 12 visual fields were evaluated in each experimental group (3 wells/group) and all GFP cells were counted. Differentiated oligodendrocyte-like cells (NG2 and APC positive) were counted separately and expressed as the percentage of total GFP cells. Every independent group was repeated for a total of three times.

### Cortical Motor evoked potentials (CMEP)

Thirty days after PBS and cells injection, 36 rats (*n* = 6 for each group, total six groups) were anesthetized with ketamine (40 mg/kg) and 1% sodium pentobarbital (30 mg/kg) and stereotaxically fixed. The sciatic nerves and sensorimotor cortex (SMC) were exposed, and then the stimulation electrodes were connected to the SMC and the recording electrodes were connected to the sciatic nerve (Supplementary Figure 3). The cortical motor evoked potentials (CMEP) were detected by the BL-420E Data Acquisition Analysis System for Life Science (Taimeng, China). The parameter setting of the CMEP signal is as follows: gain parameter 2000, time constant 0.01 s, filtering 300 Hz. To elicit a CMEP, a single pulse stimulation (50 ms in duration at a frequency of 5.1 Hz and with a 5 mV voltage density) was transmitted through the electrodes. In order to obtain high-quality waveforms for the CMEP signals, 50 CMEP responses were averaged for each rat.

### Immunohistochemical staining

Following the behavioural testing and evoked potentials recording, 48 rats of 6 groups (*n* = 8 for each group) were perfused by 4% paraformaldehyde in phosphate buffer (pH 7.4). The spinal cord was removed, post-fixed overnight in the paraformaldehyde and cryoprotected in 0.1 M PB containing 30% sucrose at 4°C. The spinal cord containing the lesion was removed and separated into rostral and caudal halves. The rostral half was for cryosectioning for cell morphology and immunohistocytochemistry, and the caudal half was for semi-thin section and also for electron microscopy. Transverse cryosections (25 μm thickness) of the spinal cord (*n* = 5 for each group) were cut and mounted on gelatin coated slides for immunostaining. Along with this, longitudinal cryosections (25 μm thickness) of spinal cord (*n* = 3 for each group) were also cut and mounted on gelatin-coated slides for immunostaining.

Primary antibodies used were as follows: mouse anti-green fluorescent protein (GFP, 1:500, Millipore, USA); mouse anti-neurofilament (NF, 1:300, Sigma, USA); rabbit anti-neurofilament (NF, 1:300, Sigma, USA); rabbit polyclonal anti-a chondroitin sulfate proteoglycan (NG2, a marker for oligodendrocyte progenitor cells, 1:200, Millipore, USA), rabbit polyclonal anti-adenomatous polyposis coli (APC, a marker for mature oligodendrocytes, 1:1000, Calbiochem); rabbit polyclonal anti-Kv1.2 (a marker for axonal Kv channel, 1:50, Alomone Labs); chicken anti-myelin basic protein (MBP, a marker for myelin, 1:500, Millipore, USA). Cy3-conjugated goat anti-mouse IgG and Cy3-conjugated rabbit anti-goat IgG (1:800, Jackson ImmunoResearch, USA), FITC-conjugated goat anti-mouse IgG and FITC-conjugated goat anti-rabbit IgG (1:200, Jackson ImmunoResearch, USA), Dylight 649-conjugated goat anti-rabbit IgG and Dylight 649-conjugated goat anti-chicken IgG (1:1000, Jackson ImmunoResearch, USA) were used as secondary antibodies.

Double immunofluorescence staining was carried out on transverse sections of spinal cord to assess the survival and differentiation of MSCs grafted. Donor MSCs were identified by a mouse anti-GFP antibody and visualized with FITC-conjugated goat secondary antibodies. Rabbit polyclonal antibodies of anti-NG2 or anti-APC were used to identify the differentiation of grafted MSCs into myelin-forming cells. Sections were washed three times with PBS and incubated with 10% normal goat serum with 0.3% Triton X-100 in PBS for 30 min at room temperature. Incubation with appropriate primary antibodies was performed overnight at 4°C. After repeated washing with PBS, sections were incubated with their respective secondary antibodies for 1 h at 37°C, washed with PBS, coverslipped, and examined under a fluorescence microscope (Leica Microsystem AG, Switzerland). Selected sections were further analyzed with a confocal Zeiss (LSM710) microscope equipped with argon/krypton laser and ZEN software.

Cell counting was conducted in 10 transverse spinal cord sections from the demyelinated spinal segment of each animal. The surviving GFP positive, NG2/GFP positive and APC/GFP positive cells were counted in 3 randomly selected unit areas (100 μm × 100 μm) in the epicentre of the demyelination/graft site of each section at 400× magnification. The number of GFP positive cells in the unit areas scrutinized was considered as the total cell number. The average number of GFP positive cells from the unit areas was designated as the number of surviving cells and used for comparison. Differentiated oligodendrocyte-like cells (NG2/GFP positive and APC/GFP positive cells) were also counted in the unit areas and expressed as a percentage of total surviving GFP positive cells for use in comparison analysis.

Triple-label immunofluorescence staining was performed in the longitudinal sections to determine the relationship of Kv channel and myelin associated with the axons in the epicentre of the demyelination/graft site. Axons were identified by a mouse anti-NF antibody. The rabbit polyclonal anti-Kv1.2 was used to identify the axonal Kv channel. The chicken anti-MBP was used to identify the myelin. Triple-label immunofluorescence staining was further analyzed with a confocal Zeiss (LSM710) microscope equipped with argon/krypton laser and ZEN software.

### Preparation of semi-thin resin sections and ultrathin sections

To quantify myelin sheaths, we adopted a method similar to that described previously^27^. Briefly, the caudal halves of the spinal cords were cut every 2 mm thickness starting from the epicentre of the demyelination/graft site to get 2 thin blocks (2 mm), and then quickly post-fixed in a fixative solution containing 2.5% glutaraldehyde for overnight at 4°C. The blocks were washed in PBS, before being placed in a 2% osmium tetraoxide (Oxkem Limited, Reading, UK) for 30 min. The blocks were washed again in PBS, and then dehydrated in a graded series of ethanol concentrations and flat-embedded in Epon 812. Semi-thin sections were cut using 6 mm glass knives on a Leica RM2065 microtome, placed on polysine slides and stained with toluidine blue (5% in a Borax solution). Finally the sections were cleared in xylene before being mounted using neutral balsam. In all groups, 4 semi-thin sections (2 sections selected from each block, 2 blocks) were selected from each animal (*n* = 5 for each group) to perform remyelination analysis. A total of 20 sections were selected from each group. Three unit areas (100 μm × 100 μm) from superficial, middle and deep of the dorsal funiculus (DF) were selected in each section and examined at 400× magnification. Three kinds of myelin sheaths, including degenerated myelin (loose, redundant sheaths that form loops), newborn myelin (remyelination; thinner sheaths and lighter in color) and normal myelin (compact, thick dark sheaths), were respectively counted within a unit area^27^. The total number of each kind of myelin sheaths from 12 unit areas per rat was calculated and compared among different groups. Ultrathin sections were prepared, stained with uranyl acetate and lead citrate, and examined under a transmission electron microscope (Philips CM10, Eindhoven, Holland).

### Immunoelectron microscopy

Three rats in the TrkC-MSCs+EA group were perfused intracardially with 0.9% saline followed by 400 ml of fixative consisting of 4% paraformaldehyde + 15% saturated picric acid + 0.1% glutaraldehyde in 0.1 M phosphate buffer at pH 7.4. The demyelinated spinal cord segments were removed, postfixed overnight at 4°C in fresh fixative without glutaraldehyde, and subsequently cut into 100 μm transverse sections on a vibratome. To improve the penetration of antibodies, vibratome sections were transferred into cryprotectant solution containing 25% sucrose and 10% glycerol in 0.1 M PBS overnight at 4°C, followed by a quick freeze-thaw in liquid nitrogen three times. After washing with TBS, the sections were treated for 1 h with 20% goat serum to block nonspecific binding of the antibody. The sections were immunostained with mouse anti-GFP antibody (1:400, Millpore, USA) for 48 h at 4°C. After washing with TBS, sections were incubated with 6 nm immunogold-labeled goat anti-mouse IgG antibody for 2 h at room temperature, and then postfixed with 2% glutaraldehyde in 0.1 M PBS for 20 min. These sections were washed, and then postfixed with 2% OsO_4_ in 0.1 M PBS, dehydrated in a graded series of ethanol concentrations, and flat-embedded in Epon 812. The tissue blocks were cut into ultrathin sections (70 nm thick) which were collected on formvar-coated nickel slot grids and stained with uranyl acetate. The sections were examined under the transmission electron microscopy (Philips CM10, Eindhoven, Holland).

### Western blot analysis

After 30 days of surgery, 30 rats (*n* = 5 for each group) were sacrificed. The spinal cord was immediately removed and the demyelinated spinal segments (0.5 cm) containing the demyelination/graft site were dissected and homogenized on ice in Western lysis buffer (Beyotime Institude of Biotechnology, China) containing 1% protease inhibitor cocktail (Sigma, USA) using homogenizers. Homogenates were centrifuged at 12,000 g for 15 min at 4°C. The supernatant liquid was collected and stored at −80°C for Western blot analysis. Equal amounts of protein (50 μg) were then loaded on a 10% SDS polyacrylamide gel (SDS-PAGE) and followed by electrophoresis for 1 h at 150 V. Proteins were transferred from the gel to a nitrocellulose membrane for 2 h at 350 mA. After blocking non-specific binding sites with 5% non-fat milk in TBST (0.5% Tween 20 in TBS) for 1 h at room temperature, the membranes were incubated with mouse anti-β-actin (1:1000, cell signaling, USA), rabbit anti- Kv1.2 (1:400, Alomone Labs) and goat anti-MBP (1:1000, Santa Cruz) overnight at 4°C, respectively. After washing, the members were treated with a horseradish peroxidase (HRP)-conjugated secondary antibody (goat anti-mouse, goat anti-rabbit or rabbit anti-goat, 1:5000, Jackson, USA) for 2 h at room temperature, and then washed repeatedly. The protein bands were visualized by enhanced chemiluminescence (ECL) detection reagents (Applygen Technologies Inc., Beijing, China), and exposed onto X-films for 1 min. The total intensities of protein bands were measured and quantified with NIH ImageJ. Background was subtracted for each band. The β-actin loading ratios were obtained by normalizing against the sham control band. The Kv1.2 channel alterations were obtained by first normalizing against their sham controls and further normalizing with the β-actin loading ratio. All Western blotting experiments were performed in triplicates.

### Statistical analysis

Data were given as the mean ± standard deviation (S.D.). They were analyzed using one-way ANOVA or repeated-measure ANOVA by statistical software SPSS17.0. The Least-Significant Difference (LSD) test was applied for post hoc test with one-way ANOVA. The significance level was set at p < 0.05. Whenever distributions failed the normality test, non-parametric tests such as MannWhitney (t-test) were used. Spearman's Rank Correlation test was used to study the correlation between different parameters.

## Supplementary Material

Supplementary Information Supplementary information
