# Supplementary material for: Combination of Electroacupuncture and Grafted Mesenchymal Stem Cells Overexpressing TrkC Improves Remyelination and Function in Demyelinated Spinal Cord of Rats
Source: Sci Rep. 2015 Mar 16;5:9133. doi: 10.1038/srep09133 (PMC5390924; doi:10.1038/srep09133)
Supplement: Supplementary Information [file srep09133-s1.pdf]

Combination of Electroacupuncture and Grafted Mesenchymal Stem Cells Overexpressing TrkC Improves Remyelination and Function in Demyelinated Spinal Cord of Rats

Ying Ding[a#], Rong-Yi Zhang[a#], Bing He[a#], Zhou Liu[a], Ke Zhang[a], Jing-Wen Ruan[e*], Eng-Ang Ling[f], Jin-Lang Wu[g], Yuan-Shan Zeng[a,b,c,d,*]

[a]Department of Histology and Embryology, Zhongshan School of Medicine, Sun Yat-sen University, Guangzhou, Guangdong 510080, China
[b]Key Laboratory for Stem Cells and Tissue Engineering Ministry of Education, Sun Yat-sen University, Guangzhou, Guangdong 510080, China
[c]Institute of Spinal Cord Injury, Sun Yat-sen University, Guangzhou, Guangdong 510127, China
[d]Co-innovation Center of Neuroregeneration, University, Nantong, Nantong, Jiangsu 226011, China
[e]Department of Acupuncture, the 1st Affiliated Hospital, Sun Yat-sen University, Guangzhou, Guangdong 510080, China
[f]Department of Anatomy, Yong Loo Lin School of Medicine, National University of Singapore, Singapore 117597, Singapore
[g]Department of Electron Microscope, Zhongshan School of Medicine, Sun Yat-sen University, Guangzhou, Guangdong 510080, China

[#] These authors contributed equally to this manuscript

* Corresponding Authors:
Yuan-Shan Zeng, M.D., Ph.D.
Department of Histology and Embryology
Zhongshan School of Medicine
Sun Yat-sen University
74# Zhongshan 2nd Road
Guangzhou 510080, China
Tel: +86-20-87332698
Fax: +86-20-87332698
E-mail address: zengysh@mail.sysu.edu.cn
or
Jing-Wen Ruan, M.D.
Department of Acupuncture
The 1st Affiliated Hospital
Sun Yat-sen University
58# Zhongshan 2nd Road
Guangzhou 510080, China
Tel: +86-20-87755766-8390
E-mail address: ruanjw@163.com

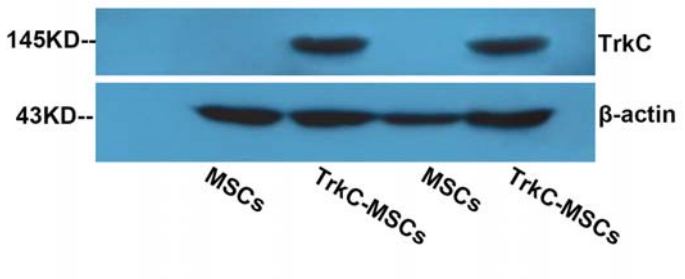

Supplementary Figure 1 | Western blot analysis of TrkC expression in cultured MSCs and Ad-TrkC transduced MSCs. Samples were run twice in the independent experiment and the experiments were repeated three times under the same experimental conditions. β-actin was used as a control.

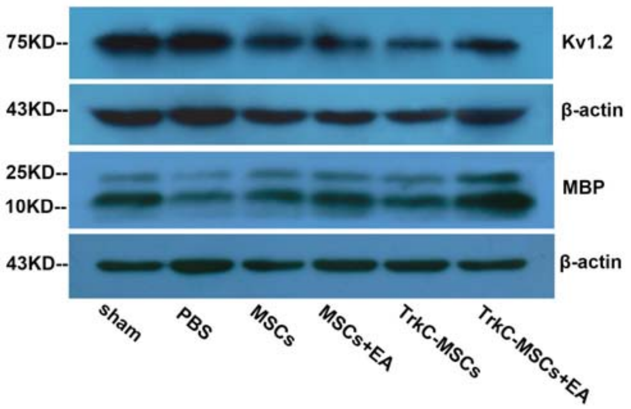

Supplementary Figure 2 | Western blot analysis of Kv1.2 and MBP expression in the sham, PBS, MSCs, MSCs+EA, TrkC-MSCs and TrkC-MSCs+EA groups. Samples were run five times in the 6 groups and the experiments were repeated twice under the same experimental conditions. β-actin was used as a control.

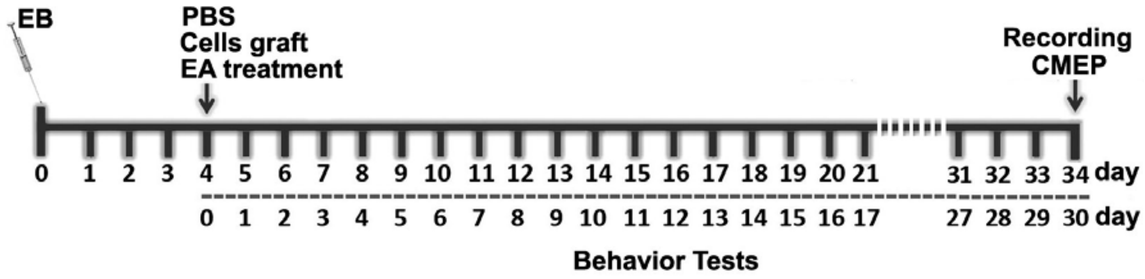

Supplementary Figure 3 | Illustrating the time points of EB injection, PBS injection, cells graft, behavior tests and recording CMEP.

Supplementary Table 1 Application of animals in different experimental procedures

Different experimental procedures	Number of rats in each group					
	sham	PBS	MSCs	MSCs +EA	TrkC-MSCs	TrkC-MSCs +EA
ELISA	5	5	5	5	5	5
Western blot	5	5	5	5	5	5
Behavioural testing; CMEPs recording; Differentiation of MSCs; Myelin counting and electron microscopy	5	5	5	5	5	5
Longitudinal sections were used to NF/Kv1.2 /MBP triple-label immunofluorescence staining.	3	3	3	3	3	3
Immunoelectron microscope	0	0	0	0	0	3
Total	18	18	18	18	18	21
